# Interleukin-1 receptor antagonist treatment in acute ischaemic stroke does not alter systemic markers of anti-microbial defence

**DOI:** 10.12688/f1000research.19308.2

**Published:** 2019-10-16

**Authors:** Laura McCulloch, Stuart M. Allan, Hedley C. Emsley, Craig J. Smith, Barry W. McColl

**Affiliations:** 1 UK Dementia Research Institute, University of Edinburgh, Edinburgh, EH16 4SB, UK; 2 Division of Neuroscience and Experimental Psychology, University of Manchester, Manchester, M13 9PT, UK; 3 Lancaster Medical School, Faculty of Health and Medicine, Lancaster University, Lancaster, LA1 4YW, UK; 4 Division of Cardiovascular Sciences, University of Manchester, Manchester, M13 9PT, UK; 5 Greater Manchester Comprehensive Stroke Centre, Manchester Centre for Clinical Neurosciences, Salford Royal NHS Foundation Trust, Salford, M6 8HD, UK

**Keywords:** stroke, IL-1Ra, immunoglobulins, complement, infection

## Abstract

**Background**: Blockade of the cytokine interleukin-1 (IL-1) with IL-1 receptor antagonist (IL-1Ra) is a candidate treatment for stroke entering phase II/III trials, which acts by inhibiting harmful inflammatory responses.  Infection is a common complication after stroke that significantly worsens outcome and is related to stroke-induced deficits in systemic immune function thought to be mediated by the sympathetic nervous system.  Therefore, immunomodulatory treatments for stroke, such as IL-1Ra, carry a risk of aggravating stroke-associated infection. Our primary objective was to determine if factors associated with antibody-mediated antibacterial defences were further compromised in patients treated with IL-1Ra after stroke.

**Methods:** We assessed plasma concentrations of immunoglobulin isotypes and complement components in stroke patients treated with IL-1Ra or placebo and untreated non-stroke controls using multiplex protein assays. Activation of the sympathetic nervous system (SNS) was determined by measuring noradrenaline, a major SNS mediator.

**Results:**  There were significantly lower plasma concentrations of IgM, IgA, IgG1 and IgG4 in stroke-patients compared to non-stroke controls, however there were no differences between stroke patients treated with placebo or IL-1Ra. Concentrations of complement components associated with the classical pathway  were increased and those associated with the alternative pathways decreased in stroke patients, neither being affected by treatment with IL-1Ra.  Noradrenaline concentrations were increased after stroke in both placebo and IL-1Ra-treated stroke patients compared to non-stroke controls.

**Conclusion:** These data show treatment with IL-1Ra after stroke does not alter circulating immunoglobulin and complement concentrations and is therefore unlikely to further aggravate stroke-associated infection susceptibility through altered availability of these key anti-microbial mediators.

## Introduction

Blocking the actions of the inflammatory cytokine interleukin-1 (IL-1) using a highly selective IL-1 receptor antagonist (IL-1Ra) reduced injury and improved outcome in multiple experimental animal models of cerebral ischemia and is in ongoing clinical stroke trials ^1– 4^. The inflammatory-modifying properties of IL-1Ra may confer protective effects to the brain after stroke; however, due to its potential for immunosuppression, it may also compromise systemic immune responses important for defence against infection. Systemic immune dysregulation is particularly important to consider in the context of stroke as patients are highly susceptible to infection, which likely involves roles for stroke-induced impairments in some immune functions ^5^. 

We have previously shown deficits in early antibody responses, particularly IgM, associated with innate-like B cells in both experimental animals and stroke patients, which may contribute to post-stroke infection susceptibility ^6^. IL-1β is reported to induce IgM production in innate-like B cells ^7^; therefore, treatment with IL-1Ra may inhibit these important anti-microbial effects. We assessed whether markers associated with antibody-mediated antibacterial defences were compromised in patients treated with IL-1Ra after stroke. In summary, our data suggest treatment with IL-1Ra is unlikely to aggravate antibody-associated immune function deficits induced by stroke.

## Methods

### Standard protocol approvals, registrations, and patient consents

This study involved tertiary analysis of plasma samples taken from a randomised, placebo-controlled phase II trial originally designed to determine the safety and biological activity of intravenous (IV) IL-1Ra ^4^. The online clinical trials registries ClinicalTrials.gov and ISRCTN went live online during the year 2000, at which time online trial registration was a relatively new recommendation. The original IV IL-1Ra trial was set-up in 2000 and commenced February 2001 and therefore this trial was not officially registered. Furthermore, tPA recanalization therapy was not approved for use in the UK prior to 2003 for patients less than 3 h from stroke onset. Recruitment to the trial closed in July 2003 prior to widespread implementation of IV alteplase in the UK ^8^. Therefore, no patients in this trial were given, or eligible for, recanalization therapy with tPA. Ethical approval for reanalysis of the samples was obtained through the Health Research Authority National Research and Ethics Service Committee (16/NW/0853). 

### Clinical trial protocol

***Participants and study procedures.*** In brief, patients ≥ 18 years of age with a clinical diagnosis of stroke within six hours of stroke onset were eligible. Exclusion criteria included National Institutes of Health Stroke Scale (NIHSS) score of ≤ 4, pre-stroke modified Rankin Scale (mRS) score of ≥ 4 or rapidly improving neurological deficit. Patients were randomly assigned to treatment with recombinant methionylated human IL-1Ra (n=17) or placebo (n=17) stratified by age (< 70 and ≥ 70 years), baseline stroke severity (NIHSS score 4–9, 10–20, ≥ 21) and time since stroke onset (< 4 or ≥ 4 h), but not by sex. IL-1Ra was initially administered as an IV loading dose of 100 mg over 60 seconds, followed by 72 hours of consecutive infusions at 2 mg/kg/h. Full patient baseline characteristics and stratification of groups are provided as *Extended data* ^9^.

Non-stroke control patients (n=13) of a similar age range with no previous history of stroke or transient ischemic attack were also recruited. Control patients were living independently at home, free of infection and able to provide written, informed consent. Controls were matched to stroke patients (six to patients receiving IL-1Ra and seven to patients receiving placebo) on a basis of age (±5 years), sex and degree of atherosclerosis, which was determined using a non-invasive assessment of either ankle-brachial pressure or carotid atherosclerosis using Doppler, as previously described ^10^.

***Blood sampling.*** Venous blood samples were collected prior to the initiation of treatment (admission), at the next 9 am time point (if admission was before 7 am or after 11 am), and then at 9 am at 24 hours, 2 days, 3 days, 4 days and at 5–7 days after stroke, into tubes containing a final concentration of 10 μg/ml pyrogen-free heparin and wrapped in cool packs. Control patients were sampled at 9 am and also at matched patient admission time (two hours) if this was not between 7 am and 11 am. Samples were centrifuged one hour after collection at 2000 *xg* for 30 min at 4°C. Plasma was separated and frozen in aliquots at -70°C until further analysis.

### Luminex analysis of immunoglobulins and complement components

Immunoglobulins and complement components were measured in plasma samples using MILLIPLEX® multiplex assays. Patient details were blinded from samples and coded samples were randomised across plates for analysis. The MILLIPLEX® _MAP_ Human Isotyping Magnetic Bead Panel- Isotyping Multiplex Assay (HGAMMAG-301K-06, Merck Millipore Corporation, Billerica, MA, USA) was used to measure IgG1, IgG2, IgG3, IgG4, IgA and IgM. MILLIPLEX® _MAP_ Human Complement Panel 1 (HCMP1MAG-19K, Merck Millipore Corporation) was used to measure C2, C4b, C5, C9, Mannose-binding lectin (MBL), Factor D (Adipsin) and Factor I. Many samples had concentrations of Factor D and Factor I below the detection range of the standard curve and so results for these analytes are not reported. MILLIPLEX® _MAP_ Human Complement Panel 2 (HCMP2MAG-19K, Merck Millipore Corporation) was used to measure C1q, C3, C3b/ iC3b, C4, Factor B, Properdin and Factor H. Samples were assayed as singlets and all samples, standards and quality controls were prepared in accordance with the manufacturer’s instructions. Samples were incubated with beads on a plate for one hour (isotyping assay) or overnight (complement assays) at 4°C and washes carried out using a magnetic plate washer. Plates were analysed using a Magpix™ Luminex® machine and Luminex xPonent® software version 4.2, with a sample volume of 50 ml per well and a minimum of 50 events counted per sample.

### Measurement of noradrenaline

Noradrenaline was measured in plasma samples using a Noradrenaline ELISA kit (BA E-5200; LDN®, Nordhorn, Germany). Patient details were blinded from samples and coded samples were randomised across plates for analysis. Samples were assayed as singlets and all samples, standards and quality controls were prepared in accordance with the manufacturer’s instructions, where noradrenaline is extracted from plasma using a cis-diol-specific affinity gel, acylated, enzymatically converted and then measured by ELISA. Optical density at 450 nm was measured using an MRX microplate Reader (Dynatech Labs, Chantilly, VA).

### Statistical analyses

All immunoglobulin and complement components were measured in μg/ml and the D’Agostino and Pearson omnibus test was used to determine Gaussian distribution of sample data. As data were non-normally distributed, sample values were log _10_-transformed. The precise kinetics of individual patient responses were varied as demonstrated by analysing when minimum and maximum values were reached in individual patients for each time point, provided as *Extended data*. To overcome this variance, the maximal and minimal concentrations of each mediator in the first seven days after stroke were compared to non-stroke controls. Maximal and minimal concentrations from IL-1Ra-treated and placebo-treated stroke patients and non-stroke controls were compared by one-way ANOVA with Bonferonni correction. Noradrenaline concentrations were measured in ng/ml and the D’Agostino and Pearson omnibus test was used to confirm Gaussian distribution of sample data. Maximal and minimal noradrenaline concentration from IL-1Ra-treated and placebo-treated stroke patients and non-stroke controls were compared by one-way ANOVA with Bonferonni correction. Each sampling time point was also compared to the admission time point using a mixed models analysis with Dunnett correction in IL-1Ra and placebo treated patients individually, provided as *Extended data*. Data analysis was performed using GraphPad Prism 8.0 statistical analysis software and for all experiments, values of *P* ≤ 0.05 were accepted as statistically significant. 

An earlier version of this article can be found on bioRxiv ( https://doi.org/10.1101/587881).

## Results

### Plasma IgM concentration is reduced after stroke and is not affected by treatment with IL-1Ra

**Figure 1.**
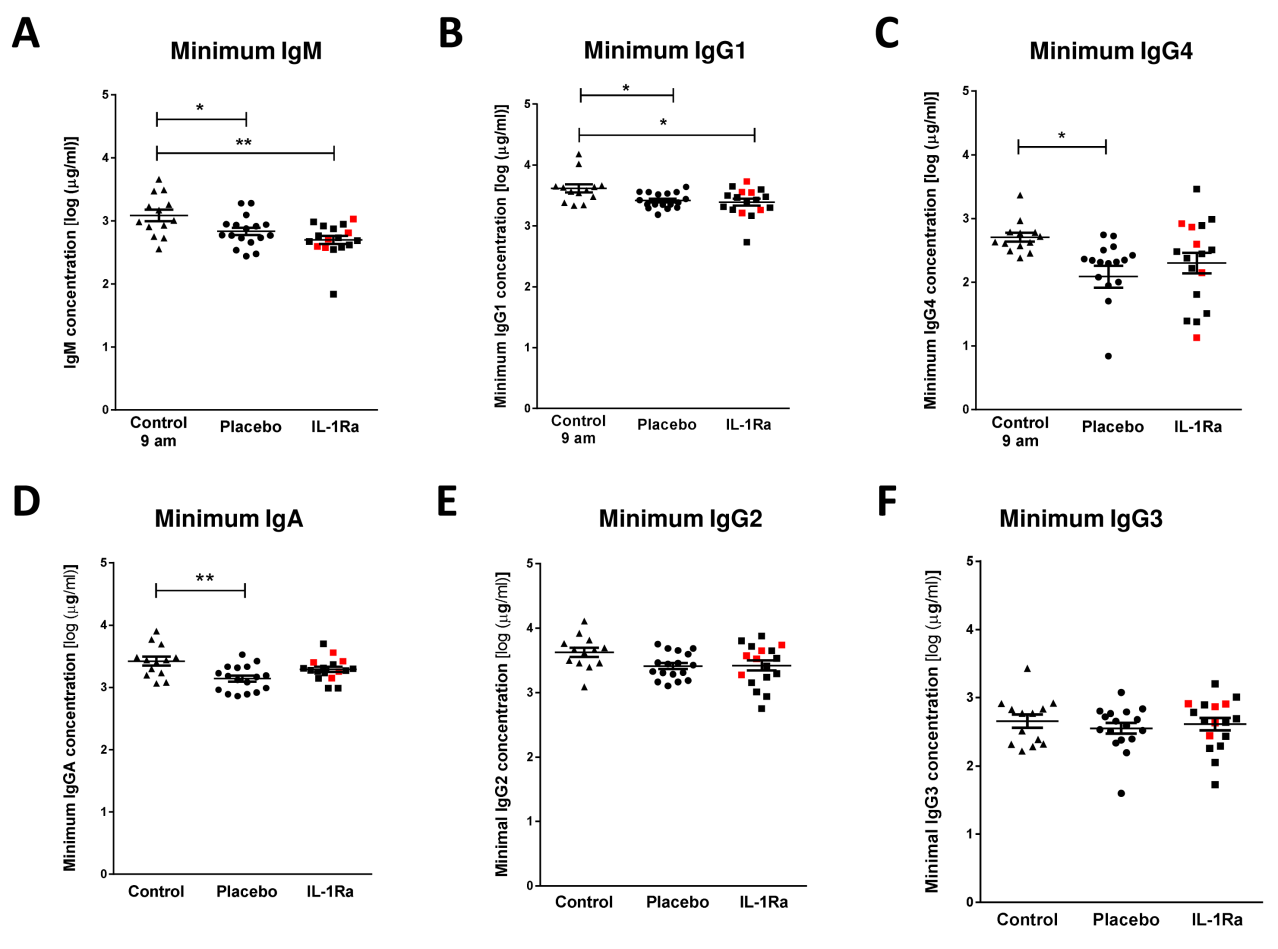
Reduced plasma IgM, IgA, IgG1 and IgG4 after stroke is not affected by IL-1 receptor antagonist (IL-1Ra). ( **A**) Minimum IgM concentration measured in the first seven days after stroke was lower in both placebo and IL-1Ra treated patients in comparison to healthy controls. Data show mean ±SD, * *P<0.05*, ** *P<0.01*, one-way ANOVA with Bonferonni correction. ( **B**) Minimum concentration of IgG1 and measured in the first seven days after stroke was reduced in both placebo and IL-1Ra treated patients in comparison to healthy controls. Minimum IgG4 ( **C**) and IgA ( **D**) concentrations were reduced in placebo-treated stroke patients in comparison to healthy controls. There was no significant difference between placebo-treated and IL-1Ra-treated stroke patients. No significant difference in IgG2 ( **E**) and IgG3 ( **F**) concentration was detected between placebo-treated and IL-1Ra-treated stroke patients in comparison to healthy controls. Data show mean ±SD, * *P<0.05*; ** *P<0.01;* one-way ANOVA with Bonferonni correction.

Immunoglobulin M (IgM) is the predominant immunoglobulin isotype associated with early B cell antibody responses to infection by innate-like B cells, which we have previously shown to be depleted after experimental stroke in mice ^6, 11, 12^. Lower minimum concentrations of IgM were measured after stroke in comparison to non-stroke controls, and no difference was found between placebo and IL-1Ra treated patients. ( Figure 1A) ^13^. Maximum IgM concentrations in the first seven days after stroke were also assessed and did not significantly differ in IL-1Ra or placebo treated patients in comparison to non-stroke controls (Supplementary Figure 1A, *Extended data*) ^9^. This indicates that the reduced minimum IgM concentration measured over the first seven days reflects an actual reduction in circulating IgM in stroke patients and is not an artefact of increased variance in IgM concentration after stroke.

### Plasma IgA, IgG1 and IgG4 concentrations are reduced after stroke and are not affected by treatment with IL-1Ra

Minimum IgG1 concentration was significantly reduced in both placebo-treated and IL-1Ra-treated stroke patients in comparison to non-stroke controls ( Figure 1B) ^13^. Minimum IgG4 ( Figure 1C) and IgA ( Figure 1D) concentrations were significantly reduced in placebo-treated stroke patients only. However, there was no significant difference in these immunoglobulins between placebo-treated and IL-1Ra-treated patients. Minimum concentrations of IgG2 ( Figure 1E) and IgG3 ( Figure 1F) were not significantly altered in IL-1Ra or placebo treated patients in comparison to non-stroke controls. Maximal circulating concentrations of all immunoglobulin isotypes measured in the first seven days after stroke were also compared to non-stroke controls and no significant differences were measured in any immunoglobulin isotypes (Supplementary Figure 1B–F, *Extended data*) ^9^. The percentage of patients reaching their minimal circulating concentration at each sampling time point was compared in placebo and IL-1Ra treated groups and showed no impact of IL-1Ra treatment on the kinetics of these responses (Supplementary Figure 5, *Extended data*).

### Concentrations of complement components are differentially affected by stroke and not affected by treatment with IL-1Ra

**Figure 2.**
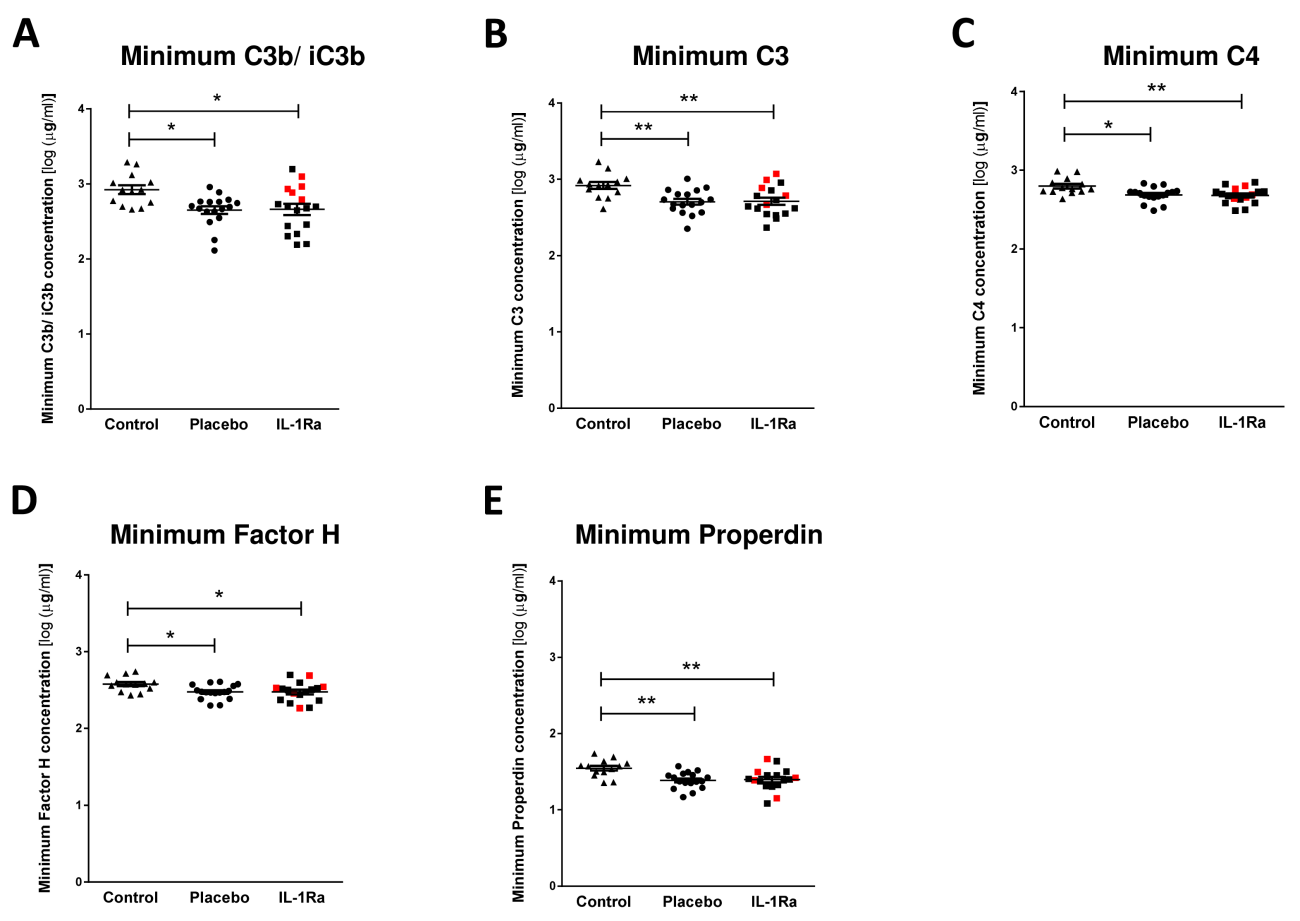
Treatment with IL-1 receptor antagonist (IL-1Ra) has no effect on complement components downregulated after stroke. Minimum concentrations of ( **A**) C3b/ iC3b, ( **B**) C3, ( **C**) C4, ( **D**) Factor H and ( **E**) Properdin were measured in the first seven days after stroke were reduced in both placebo and IL-1Ra treated patients in comparison to healthy controls. Data show mean ±SD, * *P<0.05*; ** *P<0.01;* one-way ANOVA with Bonferonni correction.

As complement components are directly associated with the antibacterial functions of immunoglobulins, we investigated stroke-induced changes in circulating complement components and if any changes observed were further influenced by treatment with IL-1Ra. Stroke induced a significant reduction in the minimum concentrations of C3b/ iC3b ( Figure 2A), C3 ( Figure 2B), C4 ( Figure 2C), Factor H ( Figure 2D) and Properdin ( Figure 2E) measured in the first seven days after stroke in both placebo and IL-1Ra treated patients in comparison to non-stroke controls ^13^. Maximum circulating concentrations of these complement components measured in the first seven days after stroke were also compared to non-stroke controls and no significant differences were seen (Supplementary Figure 2A–E, *Extended data*) ^9^. The percentage of patients reaching their minimal circulating concentration at each sampling time point was compared in placebo and IL-1Ra treated groups and showed no impact of IL-1Ra treatment on the kinetics of these responses (Supplementary Figure 6, *Extended data*).

**Figure 3.**
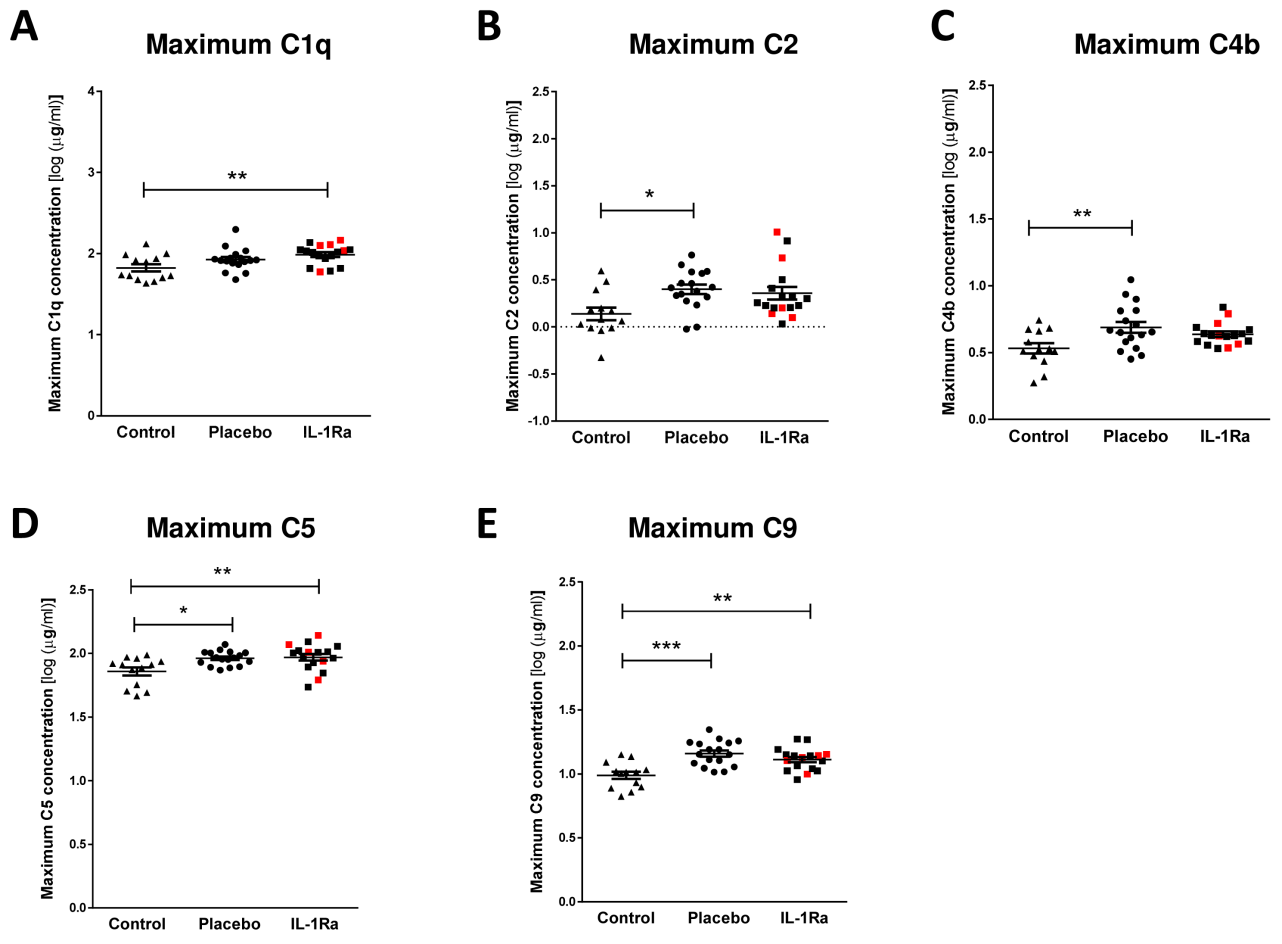
Treatment with IL-1 receptor antagonist (IL-1Ra) has no effect on complement components upregulated after stroke. Maximum concentrations of ( **A**) C1q, ( **B**) C2, ( **C**) C4b, ( **D**) C5 and ( **E**) C9 were measured in the first seven days after stroke were increased in both placebo and IL-1Ra treated patients in comparison to healthy controls. Data show mean ±SD, * *P<0.05*; ** *P<0.01;* one-way ANOVA with Bonferonni correction.

In contrast, stroke induced a significant increase in maximal circulating concentrations of C1q ( Figure 3A), C5 ( Figure 3D) and C9 ( Figure 3E) in both IL-1Ra and placebo treated patients measured in the first seven days after stroke in comparison to non-stroke controls. Maximum concentrations of C2 ( Figure 3B) and C4b ( Figure 3C) were increased in placebo-treated patients only ^13^. However, no significant difference was apparent between placebo treated and IL-1Ra treated patients for these factors, suggesting IL-1Ra treatment exerts no effects additional to stroke. Minimum concentrations of these complement components measured in the first week after stroke were also compared to non-stroke controls and no significant differences were seen (Supplementary Figure 3A–E, *Extended data*) ^9^. The percentage of patients reaching their maximum circulating concentration at each sampling time point was compared in placebo and IL-1Ra treated groups and showed no impact of IL-1Ra treatment on the kinetics of these responses (Supplementary Figure 7, *Extended data*). 

**Figure 4.**
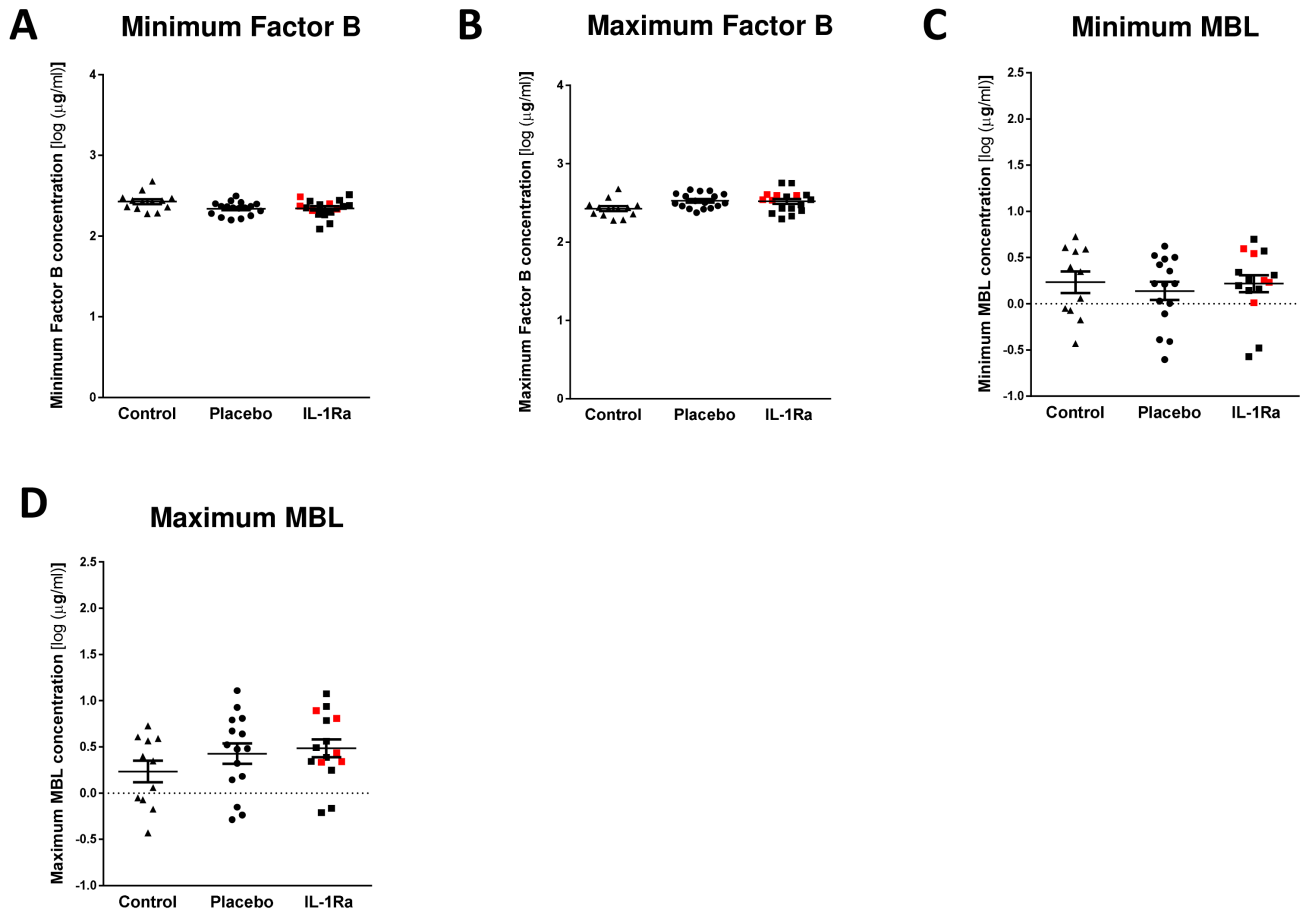
Treatment with IL-1 receptor antagonist (IL-1Ra) has no additional effect on complement components unaffected by stroke. Minimal and maximum concentrations of ( **A**, **B**) Factor B and ( **C**, **D**) mannose-binding lectin (MBL) were measured in the first seven days after stroke were unchanged in both placebo and IL-1Ra treated patients in comparison to healthy controls. Data show mean ±SD, one-way ANOVA with Bonferonni correction.

Minimal and maximal levels of Factor B, MBL and C5a measured in the first week after stroke were also compared to non-stroke controls. Concentrations of Factor B ( Figure 4A, B) and MBL ( Figure 4C, D) were not significantly altered by stroke or by treatment with IL-1-Ra ^13^. 

### Plasma noradrenaline concentration is increased after stroke and is not affected by treatment with IL-1Ra

**Figure 5.**
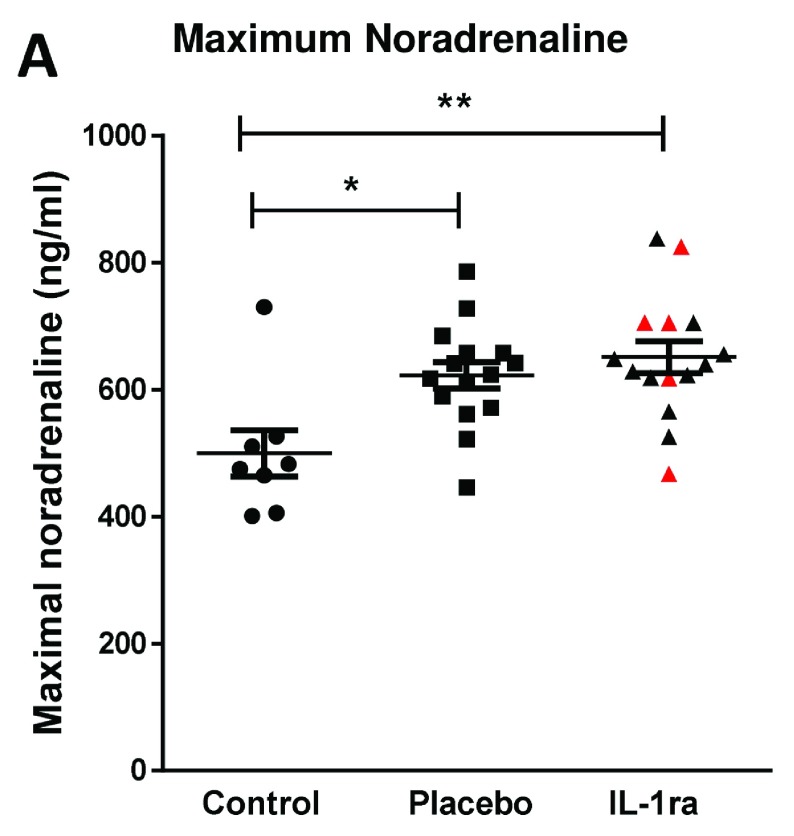
Plasma noradrenaline concentration is increased after stroke and is not affected by treatment with IL-1 receptor antagonist (IL-1Ra). Maximal noradrenaline concentration measured in the first seven days after stroke was significantly higher in both placebo and IL-1Ra treated patients in comparison to controls. Data show mean ± SD *P<0.05; **P<0.01; one-way ANOVA.

Splenic noradrenaline levels are increased after experimental stroke and may be toxic to IgM producing B cells ^6^. Maximum noradrenaline concentration measured in the first seven days after stroke was increased in both placebo and IL-1Ra treated patients in comparison to non-stroke controls ( Figure 5A) ^13^. Treatment with IL-1Ra had no additional effect on noradrenaline concentration when compared to placebo. Minimum noradrenaline concentration in the first seven days after stroke was also measured and was not significantly different to non-stroke controls (Supplementary Figure 4, *Extended data*) or affected by IL-1Ra treatment ^9^.

## Discussion

In this study, we assessed if markers associated with antibody-mediated antibacterial defences were altered in patients treated with IL-1Ra after stroke. Plasma IgM, IgG1, IgG4 and IgA immunoglobulin concentrations were reduced after stroke and this was not further altered by treatment with IL-1Ra. Assessment of complement components indicated increased concentration of complement components associated with the classical pathway of complement activation in the first week after stroke but reduced concentration of those associated with the alternative pathway without modulation by IL-1Ra. Plasma noradrenaline was increased after stroke and also not influenced by treatment with IL-1Ra. These data suggest that treatment with IL-1Ra is unlikely to aggravate antibody-associated immune function deficits induced by stroke.

The IL-1 family of cytokines play a critical role in host defence to pathogens by signalling to a variety of host cells to induce downstream effects including, but not limited to, pro-inflammatory cytokine and chemokine production, immune cell recruitment and upregulation of vascular adhesion molecules ^14, 15^. However, in conditions of sterile inflammation and tissue injury, such as stroke, these effects can aggravate primary tissue damage and impair injury repair mechanisms. Blocking IL-1 signalling has shown improved outcome in both experimental animal and patient stroke studies ^1, 4, 16^. However, the immunosuppressive effects of blocking IL-1 signalling after stroke may additionally inhibit systemic responses to infection, further increasing the risk of infection in patients who are already immune compromised ^17, 18^. Indeed, meta-analysis studies have shown an increased risk of serious infection in rheumatoid arthritis patients treated for prolonged periods with the IL-1 blocking drug anakinra ^17^. However, as of yet this has not been observed in stroke patients, potentially reflecting differences in the duration of treatment. No statistically significant differences in infection incidence were seen between IL-1Ra and placebo treated patients in this study, with 5/17 IL-1Ra treated patients experiencing infection between admission and day seven and 4/17 infections in placebo treated patients. Consistent with this pattern, we have shown here that relatively short duration of treatment with IL-1Ra after acute stroke did not further affect stroke-induced changes to circulating immunoglobulin, complement or noradrenaline concentrations and is therefore unlikely to further compromise immune defence against infection through altering bioavailability of these antibacterial mediators.

IL-1 cytokine family members are reported to have variable effects on B cell antibody production. IL-1β was reported to be important for the rapid production of anti-bacterial IgM by innate-like B cells important for early containment of infection prior to the induction of adaptive immune responses ^7, 19^. This would suggest treatment with IL-1Ra after stroke could further compromise the early production of IgM in innate-like B cells which are already known to be reduced in number after stroke ^6^. However an effect of IL-1Ra on IgM concentration was not seen. We know that experimental stroke results in a significant loss of many populations of B cells and associated IgM ^6^; therefore, it is possible that the effects of the stroke itself on B cells overwhelm any additional effects of cytokines that could moderately enhance or inhibit immunoglobulin production. Furthermore, it is not known if remaining B cells are functionally impaired and therefore able to respond to IL-1β signalling as they would under normal homeostatic conditions. We have previously reported that stroke is associated with reduced circulating IgM concentrations in comparison to non-stroke controls ^6^, an effect reproduced here. Further studies will be required to determine if IgM, or any of the mediators assessed in this study, would be useful as biomarkers to determine which patients are likely to develop infection after stroke.

We have shown for the first time that circulating IgG1, IgG4 and IgA concentrations were reduced in the first seven days after stroke in comparison to non-stroke controls. This is in agreement with previous data showing that pan-IgG concentrations were reduced in patients after stroke, although subclasses of IgG were not assessed in that study and no reduction in IgA was found at the seven day time point assessed ^20^. IgA is the most predominant immunoglobulin isotype at mucosal surfaces including the respiratory tract and is crucial for antibacterial protection at these sites ^21^. Given the early reduction of IgA in placebo-treated stroke patients, determining the effect of stroke on IgA-producing B cells at infection susceptible sites, such as the lung mucosa, could further elucidate if this has an important role in post-stroke infection susceptibility. 

In contrast to the short half-life of IgA and IgM ^21– 23^, the half-lives of IgG1 and IgG4 are reported to be 21 days and therefore, an early reduction in IgG concentration is not compatible with a lack of *de novo* production after stroke due to loss of B cells ^24^. Previous studies have suggested that reduced total-IgG after stroke may be associated with increased loss or catabolism of IgG, which could account for reductions in concentration occurring more rapidly than its natural half-life ^25^. An alternative explanation could be that reduced IgG concentration is indicative of vascular risk factors and inflammatory changes preceding stroke that are associated with stroke risk. However, control patients in this study were matched for risk factors including their degree of atherosclerosis and would be expected to show similar changes to stroke patients if these were associated with risk factors. Understanding that the kinetics of an individual immunoglobulin subset changes both preceding and as a result of stroke, and their associations with post-stroke infections, could be invaluable in providing new therapeutic targets to reduce incidence of infection and improve outcome in patients.

The complement system has a crucial role in enhancing humoral immune defence and protecting from bacterial infection via interactions with both the innate and adaptive immune systems ^26^. As activation of the complement system is closely associated with efficient immunoglobulin-mediated clearance of pathogens, we determined whether complement mediators were altered by stroke. We have assessed, for the first time, individual concentrations of multiple complement components covering all pathways of complement activation after stroke that could further compromise patient ability to fight infection. These data suggest there are no overall deficits in complement activation after stroke. Complement activation pathways converge at multiple points; however, their initial activation mechanisms are distinct. The classical complement pathway is activated when IgM or IgG immune complexes bind to C1 (composed of C1q, C1r and C1s) ^26, 27^. Maximum circulating concentration of complement components associated with the classical and lectin pathways of activation, C1q, C2 and C4b, and end stage mediators common to all pathways, C5 and C9, were increased in the first seven days after stroke in comparison to non-stroke controls. In this study, concentration of MBL was not significantly altered by stroke, however other recognition molecules such as ficolins and collectin-11 which were not analysed in this study are also important initiators of the lecting pathway of complement activation ^28^. Additionally, around 20% of the general population has a genetic deficiency in MBL associated with a circulating level of MBL <500 ng/ ml ^29^. To ensure our analysis of MBL concentration was not confounded by the inclusion of these genetic variants, patients who did not reach a circulating concentration of >500 ng/ml across the entire sampling time course were excluded from the analysis reducing our n to 15 patients per treatment group. Therefore from these data, we cannot determine the individual contribution of the classical and lectin pathway of complement activation to the increased concentration of factors common to these two pathways in the first week after stroke.

In contrast, the alternative pathway of complement activation is initiated by microbial cell surfaces and polysachharide antigen and results in a cascade that generates C3 ^26, 27^. Complement components that were significantly downregulated after stroke, C3b/ iC3b, C3, Factor H (fH) and Properdin, are more associated with the alternative pathway of complement activation. These data are in agreement with previous studies investigating systemic C-reactive protein, C3c and C4 complement concentrations in the serum of patients 24 h after ischemic stroke, which concluded that the classical pathway of complement activation was activated in the first 24 h after ischemic stroke, whereas C3c, associated with the alternative pathway, was reduced ^30, 31^. The roles of individual pathways of complement activation in infection susceptibility after stroke remain to be determined, and we note that reduced concentrations of some complement mediators may reflect pathway activation rather than suppression (e.g. via sequestration by binding to targets) therefore some caution is required in interpretation. Nonetheless, the data presented here show that IL-1Ra does not alter levels of mediators suggesting that functional effects of treatment on pathway activation or suppression are unlikely via altered bioavailability. Further more, our data suggest that overall deficits in complement concentration are unlikely to contribute to reduced antibody-mediated clearance of pathogens that may occur after stroke.

In this study, circulating noradrenaline concentrations measured in the first week after stroke were increased in comparison to non-stroke controls but were not influenced by treatment with IL-1Ra. This agrees with previous studies showing activation of the sympathetic nervous system in both stroke and subarachnoid haemorrhage patients that resulted in increased plasma noradrenaline concentrations that persisted up to 10 days ^32– 34^. Our previous studies have shown that after experimental stroke, activation of the sympathetic nervous system and release of noradrenaline within the spleen is toxic to resident B cells and preventing noradrenaline signalling using the β-blocker propranolol prevented B cell and IgM loss and resulted in reduced infectious burden ^6^. The cytokine IL-1β is also increased in the spleen after stroke and is reported to activate peripheral nerves, including the splenic nerve, and increase production of splenic noradrenaline ^35, 36^. However, blockade of IL-1β signalling did not alter circulating concentrations of noradrenaline after stroke.

There are some limitations to our study. As this was a tertiary analysis of samples collected from the original study, designed to test the safety profile and biological activity of intravenous Il-1Ra treatment after stroke, there are some caveats to the current analysis. Firstly, blood samples were collected in heparin containing tubes, whereas collection in EDTA provides optimal conditions for subsequent measurements of complement components ^37, 38^. Heparin is known to have an effect on baseline levels of complement components in serum as complement can spontaneously activate during the clotting process. However, plasma from blood samples collected in EDTA or heparin were reported to have similar baseline levels of complement components. The original sampling methods may have induced an increase in absolute measures of complement concentrations in this study, however samples were prepared as plasma, and all samples were collected by this method, any artefact of the heparin blood collection should be comparable across all samples and not affect the overall conclusions drawn from the data. 

Additionally, due to the randomisation and blinding of patients during recruitment, there was an uneven balance of haemorrhagic stroke patients versus ischemic stroke patients in the placebo and IL-1Ra treated groups (0% and 29.4% respectively, Supplementary Table 1, *Extended data*). To determine if this bias had skewed the data in the IL-1Ra treated group, we highlighted data points from haemorrhagic stroke patients in red to determine their distribution within the IL-1Ra treated group for each mediator analysed. In most cases, data points representing haemorrhagic stroke patients appeared evenly distributed throughout the data sets. Furthermore, excluding the haemorrhagic stroke patient data points from the statistical analysis did not change the interpretation of the results for each mediator. This suggests that the uneven representation of haemorrhagic stroke patients in the IL-1Ra treated group did not impact the overall conclusions in this study. However studies designed to investigate the pathophysiology of immune suppression after stroke in different stroke subsets would be required to fully understand the extent to which stroke subtype can impact the extent of changes to the immune system.

In summary, we have shown that treatment with IL-1Ra after stroke does not affect circulating concentrations of immunoglobulins, complement components or noradrenaline and is therefore unlikely to further increase patient susceptibility to infection via pathways in which these mediators are key participants. This aligns with data from IL-1Ra Phase II trials, in which treatment of stroke patients with IL-1Ra did not aggravate incidence of infection ^4, 13^. These data suggest that blocking IL-1 for short durations as applied in an acute stroke context may be relatively safe from the perspective of infection risk. Additionally, the treatment-independent reductions in circulating immunoglobulin concentrations detected after stroke in this study further support that antibody mediated immune defence may be an important therapeutic target for immunomodulation aimed at reducing the burden of infection after stroke. 

## Data availability

### Underlying data

Edinburgh Data Share: Interleukin-1 receptor antagonist treatment in acute ischaemic stroke does not alter systemic markers of anti-microbial defence. https://doi.org/10.7488/ds/2626

This project contains the following underlying data:

- Full noradrenaline data by patient and timepoint.xlsx (noradrenaline concentrations in ng/ml)
- Full complement data by patient and timepoint.xlsx (complement concentrations in μg/ml)
- Full immunoglobulin data by patient and timepoint.xlsx (immunoglobulin concentrations in μg/ml)
- Figure 1A dataset.pzf – Figure 5A dataset.pzf (raw data underlying Figure 1– Figure 5 in GraphPad Prism format)
- Figure 1 full data excel format.xlsx (minimum concentrations of immunoglobulins in μg/ml)
- Figure 2 full data excel format.xlsx (minimum concentrations of complement components C3b/iC3b, C3, C4, Factor H and Properdin in μg/ml)
- Figure 3 full data excel format.xlsx (maximum concentrations of complement components C1q, C2, C4b, C5 and C9 in μg/ml)
- Figure 4 full data excel format.xlsx (concentrations of complement components Factor B and MBL in μg/ml)
- Figure 5 full data excel format.xlsx (maximum concentrations of noradrenaline in ng/ml)

### Extended data

Edinburgh Data Share: Supplementary Figures: Interleukin-1 receptor antagonist treatment in acute ischaemic stroke does not alter systemic markers of anti-microbial defence. https://doi.org/10.7488/ds/2626 ^9^

This project contains the following extended datain the zip folder “SupplementaryData”:

- Supplementary Figure 1.tif (figure showing maximum concentrations of immunoglobulin)
- Supplementary Figure 2.tif (figure showing maximum concentrations of complement components C3b/iC3b, C3, C4, Factor H and Properdin)
- Supplementary Figure 3.tif (figure showing minimum concentrations of complement components C1q, C5, C9, C2 and C4b)
- Supplementary Figure 4.tif (figure showing minimum concentrations of noradrenaline)
- Supplementary Table 1.tif (table of baseline characteristics of stroke patients and stratification of groups)
- Supplementary Figure 5.tif (figure showing the percentage of patients reaching minimum circulating concentration of immunoglobulin subsets at each sampling time point)
- Supplementary Figure 6.tif (figure showing the percentage of patients reaching minimum circulating concentration of complement components reduced by stroke at each sampling time point)
- Supplementary Figure 7.tif (figure showing the percentage of patients reaching maximum circulating concentration of complement components increased by stroke at each sampling time point)
- Supplementary Figure 8.tif (figure showing immunoglobulin concentration in IL-1ra and placebo treated patients at individual sampling points after stroke)
- Supplementary Figure 9.tif (figure showing alternative pathway complement concentration in IL-1ra and placebo treated patients at individual sampling points after stroke)
- Supplementary Figure 10.tif (figure showing classical pathway complement concentration in IL-1ra and placebo treated patients at individual sampling points after stroke)
- Supplementary Figure 11.tif (figure showing complement concentration in IL-1ra and placebo treated patients at individual sampling points after stroke)
- Data Supplementary Fig 1.xlsx - Data Supplementary Fig 4.xlsx (data underlying Supplementary Figures 1-4)
- Data Supplementary Fig 5.xlsx (Supplementary Table 1 in spreadsheet format)
- Complement time course data Supplementary figures 6, 7, 9, 10, 11.xlsx (data underlying supplementary figures 6, 7, 9 10 and 11)
- Immunoglobulin time course data Supplementary figures 5, 8.xlsx (data underlying supplementary figures 5 and 8)

Data are available under the terms of the Creative Commons Attribution 4.0 International license (CC-BY 4.0).
