# Supplementary material for: Interleukin-1 receptor antagonist treatment in acute ischaemic stroke does not alter systemic markers of anti-microbial defence
Source: F1000Res. 2019 Oct 16;8:1039. Originally published 2019 Jul 10. [Version 2] doi: 10.12688/f1000research.19308.2 (PMC6820822; doi:10.12688/f1000research.19308.2)
Supplement: Peer review history

# Peer review: Reviewer response for version 2

**DOI:** 10.5256/f1000research.23010.r55271

**Published:** 2019-10-28

**Authors:** Stefano Fumagalli

**Affiliations:** 1 Department of Neuroscience, Mario Negri Institute for Pharmacological Research, IRCCS, Milan, Italy

I am happy with this revised version of the article. As a minor comment, I would suggest to state in figure legends, besides in discussion, that the red dots in the graphs represent hemorrhagic patients.

I confirm that I have read this submission and believe that I have an appropriate level of expertise to confirm that it is of an acceptable scientific standard.

# Peer review: Reviewer response for version 1

**DOI:** 10.5256/f1000research.21167.r51311

**Published:** 2019-07-26

**Authors:** Stefano Fumagalli

**Affiliations:** 1 Department of Neuroscience, Mario Negri Institute for Pharmacological Research, IRCCS, Milan, Italy

McCulloch *et al. *addressed whether stroke patients treated with IL-1 receptor antagonist (IL-1Ra) had factors associated with antibody-mediated antibacterial defences compromised. Based on their results, the authors conclude that IL-1Ra does not alter circulating immunoglobulin and complement factors in patients’ blood, thus the treatment is not expected to further aggravate stroke-associated infection.

 The addressed issue is of pivotal importance for stroke patient treatment, especially for treatment protocols using anti-inflammatory agents. Post-treatment complications have contributed to the unsuccessful clinical translation of promising anti-inflammatory compounds that proved effective in experimental models. 

 In its present form, the work presents some weakness that the authors should tackle.

  

1. Data are presented as the minimum or maximum levels of immunoglobulins and complement factors observed in a 7 day-period after stroke. Blood was sampled at admission (prior to treatment) and at 1, 2, 3, 4 and 5-7 days after stroke. I think that, being so many sampling times available, and post-stroke inflammation quickly changing over time, a picture of the temporal changes in treated vs. non-treated patients would be very informative. As such, I suggest:
  - to add the information on when min (or max) concentrations were reached in each condition, comparing them;
  - to add a statistical analysis for repeated measures comparing IL-1Ra patients’ sampling at each time after treatment vs. admission (prior to treatment).
2. Had patients received recanalization therapies? Since the temporal window of IL-1Ra for some patients is still within that of thrombolytic therapies (<4.5h), the authors should explain why they did not receive them if so. From what I can understand, the specimens used come from a trial registered in 2000, thus likely explaining the use of the placebo in patients eligible for recanalization therapies according to present guidelines. I think that this needs clarification in the text.
3. There is a big difference in placebo vs. IL-1Ra treated patients in the frequency of haemorrhagic stroke (0% vs. 29.4%). Did the authors consider to correct for this potentially impacting difference?
4. The measure of complement components may be biased. Indeed blood specimens were collected in the presence of heparin, which does not represent the standard for running unbiased complement protein analysis. Complement proteins and activation products should be measured in blood sampled in the presence of EDTA (Bergseth *et al.*, 2013 ^1^).
5. In complement system biology, the decrease of circulating levels of recognition molecules in acute phases is generally regarded as an index of complement activation (through consumption of the circulating proteins binding their targets). On later times, circulating levels also mirror increased synthesis and they may increase. This should be considered when discussing the observations relative to complement measures. Moreover, complement system activation in stroke patients is reported in the first 6h (Zangari *et al.*, 2016 ^2^), therefore a comparison of admission vs. later sampling in IL-1Ra treated patients would be informative (do they recover to control physiological complement protein levels as expected?).
6. There is now a general consensus that the lectin pathway of complement activation is that mainly involved after ischemic stroke. This has been reviewed in Orsini *et al.* (2014 ^3^) and Fumagalli and De Simoni (2016 ^4^). Besides MBL, the lectin pathway comprises other recognition molecules such as ficolins and collectin-11, that play a major role in ischemic patients (especially ficolin-1 and ficolin-3; refer to the above mentioned reviews). In this regard the conclusions drawn by the authors that: ‘As concentration of MBL itself was not significantly altered by stroke, this suggests the classical complement activation is specifically activated after stroke’ sounds incorrect. Of note, MBL concentrations are genetically determined and about 20% of the general population bears variants causing MBL deficiency (Garred *et al.*, 2006 ^5^). Indeed in figure 4C the authors show MBL levels below the detection limit. It is acknowledged that a circulating level of MBL < 500 ng/mL is associated with MBL genetic variants (Eisen *et al.*, 2008 ^6^). Thus it is not surprising that no MBL changes were observed. A more clear analysis could be done excluding the MBL-deficient patients. Interestingly, these latter patients develop less ischemic lesion volume and deficits than those with normal MBL circulating levels (Osthoff *et al.*, 2011 ^7^), thus further supporting lectin pathway critical contribution to stroke pathophysiology.

I confirm that I have read this submission and believe that I have an appropriate level of expertise to confirm that it is of an acceptable scientific standard, however I have significant reservations, as outlined above.

## References

[1] : An international serum standard for application in assays to detect human complement activation products. *Mol Immunol*.2013;56(3) : 10.1016/j.molimm.2013.05.221 232-9 10.1016/j.molimm.2013.05.221 23787367

[2] : Early ficolin-1 is a sensitive prognostic marker for functional outcome in ischemic stroke. *J Neuroinflammation*.2016;13: 10.1186/s12974-016-0481-2 16 10.1186/s12974-016-0481-2 26792363PMC4721111

[3] : Versatility of the complement system in neuroinflammation, neurodegeneration and brain homeostasis. *Front Cell Neurosci*.2014;8: 10.3389/fncel.2014.00380 380 10.3389/fncel.2014.00380 25426028PMC4224073

[4] : Lectin Complement Pathway and Its Bloody Interactions in Brain Ischemia. *Stroke*.47(12) : 10.1161/STROKEAHA.116.012407 3067-3073 10.1161/STROKEAHA.116.012407 27811336

[5] : Mannose-binding lectin and its genetic variants. *Genes Immun*.2006;7(2) : 10.1038/sj.gene.6364283 85-94 10.1038/sj.gene.6364283 16395391

[6] : Low serum mannose-binding lectin level increases the risk of death due to pneumococcal infection. *Clin Infect Dis*.2008;47(4) : 10.1086/590006 510-6 10.1086/590006 18611155PMC7107952

[7] : Mannose-binding lectin deficiency is associated with smaller infarction size and favorable outcome in ischemic stroke patients. *PLoS One*.2011;6(6) : 10.1371/journal.pone.0021338 e21338 10.1371/journal.pone.0021338 21712986PMC3119675

# Response

**Published:** 2019-10-07

**Authors:** Laura McCulloch

**Affiliations:** University of Edinburgh, UK

We are pleased the reviewer recognises the relevance of this study to the future treatment of stroke patients and thank them for their insightful and constructive comments which we have addressed as detailed below.  We hope that these changes meet with the reviewer’s approval.

**1. Data are presented as the minimum or maximum levels of immunoglobulins and complement factors observed in a 7 day-period after stroke. Blood was sampled at admission (prior to treatment) and at 1, 2, 3, 4 and 5-7 days after stroke. I think that, being so many sampling times available, and post-stroke inflammation quickly changing over time, a picture of the temporal changes in treated vs. non-treated patients would be very informative. As such, I suggest:**

**- to add the information on when min (or max) concentrations were reached in each condition, comparing them;**

**- to add a statistical analysis for repeated measures comparing IL-1Ra patients’ sampling at each time after treatment vs. admission (prior to treatment).**

We appreciate that there are limitations in analysing the data as the minimum or maximum concentration reached over a sampling time course.  However variation in the kinetics of individual patient immune responses to stroke can be affected by many factors including stroke severity, presence of infection and timing of infection onset. In a small patient cohort as reported here (n=17), this variation can have a large impact at individual sampling time points. Assessing minimal or maximal concentrations over a relatively short period of time (e.g. 1 week) within which there are multiple sampling time points can enable discovery of temporal responses to stroke that that are biologically meaningful but where the peaks/troughs may be occurring at slightly different time points in individual patients. 

We do agree, however, that it is important to ensure more granular temporal patterns are not missed. To ensure this, we have analysed the time points at which minimal or maximal concentrations of mediators significantly changed by stroke were reached, as suggested by the reviewer. The percentage of patients reaching their minimum circulating concentration of immunoglobulins ( **Supplementary Figure 5, *Extended Data***) and minimum ( **Supplementary Figure 6, *Extended Data***) or maximum circulating concentration of complement components ( **Supplementary Figure 7, *Extended Data***) at each time point was compared in placebo and IL-1ra treated groups.  These analyses have been added to the relevant results sections (Results, lines 187-190, 203-206, and 217-220). No differences in kinetics were apparent in IL-1Ra versus placebo treated groups further confirming the key findings that IL-1Ra has no significant additional effect on these mediators over and above the effects of stroke.  This analysis has been described in the manuscript in Methods section under Statistical analyses section, lines 145-148.

We thank the reviewer for the suggestion to include some statistical analyses to account for the repeated sampling that occurred in individual patients.  As there were missing samples in a few patients, a mixed effects model with Dunnett’s post-test was used to analyse each sampling time point compared to admission in both IL-1Ra treated patients, as suggested by the reviewer, and placebo treated patients.  As expected, due to the variance when minimum and maximum concentrations were reached in individual patients, there were few significant changes at individual time points in comparison to admission ( **Supplementary Figure 9-12**, ***Extended data***).  However, many immunoglobulin subsets were significantly reduced at d4 post stroke, and at some other sampling time points, in both IL-1Ra and placebo treated patients showing that the loss of immunoglobulins after stroke can still be detected in a small cohort with high variance in the kinetics of individual responses ( **Supplementary Figure 8**, ***Extended data***).  This reanalysis has been acknowledged in the manuscript in Methods section, under statistical analyses.  Again, there were no major differences in responses between IL-1ra and placebo treated patients, with the exception of IgM which showed significantly reduced concentrations at d4 In the IL-1Ra treated group but not in the placebo treated group.  Overall, these analyses further confirm the overall conclusion that IL-1Ra does not exacerbate changes to circulating immune mediators induced by stroke. 

**2. Had patients received recanalization therapies? Since the temporal window of IL-1Ra for some patients is still within that of thrombolytic therapies (<4.5h), the authors should explain why they did not receive them if so. From what I can understand, the specimens used come from a trial registered in 2000, thus likely explaining the use of the placebo in patients eligible for recanalization therapies according to present guidelines. I think that this needs clarification in the text.**

The samples used in this study were tertiary analysis of plasma samples taken from a randomised, placebo-controlled phase II trial originally designed to determine the safety and biological activity of intravenous (IV) IL-1Ra and recruitment for this study began in 2001, completing in 2003.  tPA was not approved in the UK for treatment for patients <3 h from stroke onset until 2003 and for patients 3- 4.5 h from onset until 2012, therefore recanalization therapies were not an option for patients in this original study.  We thank the reviewer for raising this important point and have provided details in the manuscript (Methods section Standard protocol approvals, registrations and patient consents lines 76-80).

**3. There is a big difference in placebo vs. IL-1Ra treated patients in the frequency of haemorrhagic stroke (0% vs. 29.4%). Did the authors consider to correct for this potentially impacting difference?**

Due to the blinding and randomisation of patient recruitment in the original study all haemorrhagic stroke patients were by chance allocated to the IL-1Ra treatment group.  We agree with the reviewer that as little is known about the differential effects of haemorrhagic versus ischemic stroke on the immune system, the frequency of haemorrhagic strokes in the IL-1Ra treatment group may have the potential to bias the data.  To address this, we identified and highlighted the data points that represent haemorrhagic stroke patients in each mediator analysed in the main figures. The data points representing the minimum circulating concentrations of immunoglobulins measured in the first week after stroke in haemorrhagic stroke patients were distributed throughout the range of concentrations measured in the IL-1Ra treated group suggesting that in this data set, the high proportion of haemorrhagic stroke patients in one treatment group did not skew the interpretation of these results ( **Figure 1**).  A similar distribution of haemorrhagic stroke patients was apparent in the majority of complement components significantly reduced ( **Figure 2**), increased ( **Figure 3**) or unchanged ( **Figure 4**) and in increased noradrenaline levels ( **Figure 5**) measured in the first week after stroke.  The exception to this was in the minimum concentrations of C3b/ iC3b and C3 ( **Figure 2A and 2B**) where haemorrhagic stroke patient data points localised to the higher end in the range of measured minimum circulating concentrations within the IL-1Ra treated group.  However, when data points from haemorrhagic stroke patients were excluded from the IL-1Ra treated data set and statistical analysis was performed, there were no significant differences in minimal circulating concentrations of C3b/ iC3b or C3 between the IL-1Ra and placebo treated groups.  Indeed, reanalysis of all mediators excluding the haemorrhagic stroke patient data points did not alter the statistical results or conclusions drawn in the results section.

We have amended our discussion to acknowledge the greater frequency of haemorrhagic stroke patients within the IL-1ra treated patient group, highlight the reanalysis we have performed and confirm that future experiments designed to determine if differences exist between immune responses to ischemic versus haemorrhagic stroke are required to further our understanding of post stroke immune suppression (Discussion).

**4.  The measure of complement components may be biased. Indeed blood specimens were collected in the presence of heparin, which does not represent the standard for running unbiased complement protein analysis. Complement proteins and activation products should be measured in blood sampled in the presence of EDTA (Bergseth *et al.*, 2013** **^1^** **).**

The blood samples were collected in heparin containing tubes for the original study, whereas collection in EDTA would have provided optimal conditions for subsequent measurements of complement components ^1, 2^.  Heparin is known to have an effect on baseline levels of complement components in serum as complement can spontaneously activate during the clotting process.  However, plasma from blood samples collected in EDTA or heparin were reported to have similar baseline levels of complement components ^1^.  There may be a minimal increase in absolute measures of complement concentrations in the samples used in this study due to original sample collection methods. However as all plasma samples were collected by this method, any artefact of the heparin collection should be comparable across all samples and therefore not alter our conclusions on the effects of stroke on complement components in the manuscript.  We have acknowledged this caveat of the study in our discussion and thank the reviewer for this comment (Discussion).

**5. In complement system biology, the decrease of circulating levels of recognition molecules in acute phases is generally regarded as an index of complement activation (through consumption of the circulating proteins binding their targets). On later times, circulating levels also mirror increased synthesis and they may increase. This should be considered when discussing the observations relative to complement measures. Moreover, complement system activation in stroke patients is reported in the first 6h (Zangari *et al.*, 2016** **^2^** **), therefore a comparison of admission vs. later sampling in IL-1Ra treated patients would be informative (do they recover to control physiological complement protein levels as expected?).**

As reported in response to point 1 from the reviewer, individual patient trajectories were highly varied with patients reaching their maximal or minimal concentrations at various time points from admission to 5-7 d ( *Extended data*, Supplementary Figures 7 and 8). Due to this variance, no individual time points after stroke showed significantly different concentrations of any complement component in comparison to control samples, including the later 5-7 d sampling time point.  Our analysis has detected common changes to complement components after stroke across this time window.  We agree with the reviewer our method limits the capacity to discuss these findings in terms of activation of complement pathways as we cannot associate these changes to a specific time post stroke or post infection.  We have altered our discussion of the data to remove any speculation about activation or suppression of complement pathways and instead have reported the overall changes in concentration and their relationship to the different arms of the complement cascade only and have added information that reduced concentration of some complement mediators may reflect pathway activation through sequestration (Discussion).  We have acknowledged that to determine if the changes to complement components were associated with increased early activation or increased synthesis of components, further studies would be required in a larger patient cohort with more stringent inclusion and exclusion criteria to account for the variance in the kinetics of individual patient responses (Discussion).

**6. There is now a general consensus that the lectin pathway of complement activation is that mainly involved after ischemic stroke. This has been reviewed in Orsini et al. (20143) and Fumagalli and De Simoni (20164). Besides MBL, the lectin pathway comprises other recognition molecules such as ficolins and collectin-11, that play a major role in ischemic patients (especially ficolin-1 and ficolin-3; refer to the above mentioned reviews). In this regard the conclusions drawn by the authors that: ‘As concentration of MBL itself was not significantly altered by stroke, this suggests the classical complement activation is specifically activated after stroke’ sounds incorrect. Of note, MBL concentrations are genetically determined and about 20% of the general population bears variants causing MBL deficiency (Garred et al., 20065). Indeed in figure 4C the authors show MBL levels below the detection limit. It is acknowledged that a circulating level of MBL < 500 ng/mL is associated with MBL genetic variants (Eisen et al., 20086). Thus it is not surprising that no MBL changes were observed. A more clear analysis could be done excluding the MBL-deficient patients. Interestingly, these latter patients develop less ischemic lesion volume and deficits than those with normal MBL circulating levels (Osthoff et al., 20117), thus further supporting lectin pathway critical contribution to stroke pathophysiology.**

We thank the reviewer for this insightful comment.  In response to this we have reanalysed our data to determine which patients in the study did not reach a circulating level of >500 ng/ml at any sampling time point and excluded these patients from our analysis of MBL concentration.  We identified two patients in each of the control, IL-1Ra and placebo groups that met this criteria and reanalysed our minimum and maximum circulating concentration of MBL with these patients excluded ( **Figure 4**).  Remaining data points on this graph that are <500 ng/ ml were from patients whose minimum circulating MBL level fell to this value, but had read at >500 ng/ ml at other sampling time points, preventing the possibility that they had a genetic MBL deficiency.  With the exclusion of these patients there were still no significant differences in maximum or minimum MBL concentration measured in the first week after stroke in placebo or IL-1Ra treated patients.  The potential for these genetic variants to confound our analysis and the results of our reanalysis excluding these patients was added to the discussion. However as we have not measured other components specific to the lectin pathway of complement activation we have amended the manuscript to show that we cannot draw any conclusions on the effect of stroke on the lectin pathway from the data within this manuscript (Discussion).

1.            Mollnes TE, Garred P, Bergseth G. Effect of time, temperature and anticoagulants on in vitro complement activation: consequences for collection and preservation of samples to be examined for complement activation. Clinical and experimental immunology 1988;73:484-488.

2.            Bergseth G, Ludviksen JK, Kirschfink M, Giclas PC, Nilsson B, Mollnes TE. An international serum standard for application in assays to detect human complement activation products. Molecular Immunology 2013;56:232-239.

# Peer review: Reviewer response for version 1

**DOI:** 10.5256/f1000research.21167.r51097

**Published:** 2019-07-22

**Authors:** María Ángeles Moro

**Affiliations:** 1 Neurovascular Research Unit, Department of Pharmacology, Faculty of Medicine, Instituto de Investigación Hospital 12 de Octubre (i+12), Complutense University of Madrid, Madrid, Spain

The beneficial properties of IL-1ra have been widely demonstrated in numerous experimental models of cerebral ischemic injury. For this reason, the molecule is currently under evaluation in ongoing clinical trials. However, given the mechanism of action of IL-1ra, possible adverse effects of this drug and derived from its immunosuppressive action, such as the potentiation of stroke-related infections, might hamper its clinical use. In this context, the aim of the work by McCulloch *et al.* is highly relevant by trying to ascertain whether factors associated with enhanced susceptibility to bacterial infections are increased in IL-1ra-treated patients after stroke. The authors explored a wide array of very well selected parameters, ranging from Ig isotypes, components of the complement system, up to noradrenaline, as a parameter of the stress response. The results show that whereas these factors, as expected, are affected by stroke, none of them was affected by the treatment, clearly supporting that, at least with the protocol of administration used for this study, IL-1ra is safe as regards to immunosuppression-derived complications.

I confirm that I have read this submission and believe that I have an appropriate level of expertise to confirm that it is of an acceptable scientific standard.
